# Assessing the Plastisphere from Floating Plastics in the Northwestern Mediterranean Sea, with Emphasis on Viruses

**DOI:** 10.3390/microorganisms12030444

**Published:** 2024-02-22

**Authors:** Ana Luzia Lacerda, Jean-François Briand, Véronique Lenoble, Eliézer Quadro Oreste, Felipe Kessler, Maria Luiza Pedrotti

**Affiliations:** 1 Laboratoire d’Océanographie de Villefranche-sur-Mer (LOV), CNRS, UMR 7093, Sorbonne Université, 06230 Villefranche-sur-Mer, France; maria-luiza.pedrotti@imev-mer.fr; 2 Laboratoire MAPIEM, EA 4323, Université de Toulon, 83041 Toulon, France; jean-francois.briand@univ-tln.fr; 3 Institut Méditerranéen d’Océanologie (MIO), CNRS, IRD, MIO, Université de Toulon, Aix Marseille University, 83041 Toulon, France; lenoble@univ-tln.fr; 4 Escola de Química e Alimentos (EQA), Campus Carreiros, Av Itália, Federal University of Rio Grande (FURG), Rio Grande 96203-000, Brazil; eliezerquadro@gmail.com (E.Q.O.); felipekessler@gmail.com (F.K.)

**Keywords:** plastics, marine environment, biofilm, bacteria, eukaryotes, viruses, pathogens

## Abstract

Plastics in the ocean create the “plastisphere”, a diverse habitat hosting various life forms. Other than the pollution induced by plastics, the co-occurrence of primary producers, symbiotic organisms, decomposers, and pathogens within the plastisphere raises questions about how they influence the dynamics of marine ecosystems. Here, we used a shotgun DNA-sequencing approach to describe the species thriving on floating plastics collected in two Mediterranean sites. Our findings revealed many species of bacteria, eukaryotes, viruses, and archaea on each plastic. Proteobacteria was dominant (70% of reads in the entire dataset), with other groups such as Ascomycota fungi (11%) and Bacteroidetes (9%) also being represented. The community structure was not affected by the polymeric composition or the plastic shape. Notably, pathogenic *Vibrio* species, including *V. campbelli*, *V. alginolyticus*, and *V. coralliilyticus*, were among the most abundant species. Viruses, despite showing lower relative abundances, occurred in all samples, especially Herpesvirales, Caudovirales, and Poxviridae groups. A significant finding was the presence of the White Spot Syndrome virus (WSSV). This pathogen, responsible for devastating outbreaks in aquaculture systems, had not been previously reported in the marine plastisphere. Our study emphasizes the need for further investigation into the ecological and economic impacts of plastisphere organisms in the ocean.

## 1. Introduction

Plastics, known for their long-lasting nature and presence in terrestrial, aquatic, and aerial habitats, pose a significant threat to our planet. This is a transboundary problem contributing to the triple planetary crises of climate change, pollution, and biodiversity loss currently faced by humanity [1]. The additional alarming fact is that 99% of plastics and their associated chemicals are made of fossil fuel [2], resulting in substantial greenhouse gas (GHG) emissions during the whole plastic cycle. In addition, extreme events as a consequence of climate change (e.g., storms and sea level rise) increase the spread of plastics among terrestrial, freshwater, marine and atmospheric environments [3]. Furthermore, the increase in the ocean temperature may facilitate the settling and distribution of alien and invasive species [4], including those living attached to marine plastic debris.

The polymeric nature of plastics, coupled with the various substances they can adsorb (e.g., additives, metals, and persistent organic pollutants), make plastics a unique substrate for microbial attachment in the ocean [5]. As plastics are relatively new and artificial substrates in marine systems, they may break natural boundaries and disturb the ecosystem functioning [6]. For example, recent studies have shown that the plastisphere can serve as a reservoir and favor the appearance of antibiotic-resistant genes (ARG) [7]. In addition, organisms that already have ARGs may have the opportunity to colonize plastic substrates in the ocean. These ARGs can potentially be transferred within the plastisphere and favor the spread of pathogens in different environmental compartments, thus increasing the risks of treatment failures, and this could be an issue for both environmental and human health [8,9].

While the adverse effects of plastics in the ocean have been extensively documented, such as the ingestion and entanglement of marine organisms, which impact over 1200 taxa [10], the role of these materials as artificial substrates in the development of complex and self-sustaining communities [11,12] have gained more attention in the last decade. Nonetheless, there are still significant knowledge gaps regarding the diversity of organisms inhabiting plastics in the ocean, as well as their ecological role. In fact, the majority of studies in this field were conducted after the term “plastisphere” was proposed, referring to these communities associated with plastics in the ocean [13]. The concept of the plastisphere was expanded to encompass the entire distribution of plastic-associated organisms in freshwater [14] and terrestrial habitats [15]. The DNA-metabarcoding approach has revolutionized the study of plastisphere communities by enabling the identification of multiple microbial taxa with high sensitivity, including those that are rare or present at low abundance levels [16]. This has provided an understanding of their community structure, with various taxa possessing different ecological functions, including saprotrophs, primary producers, symbionts, and parasites [17]. 

While bacterial communities have been extensively studied in the marine plastisphere, other groups such as eukaryotes [17,18] and, especially, viruses [19,20] have received significantly less attention. It is pivotal to understand the full composition of plastisphere communities in order to investigate the relationships and impacts these organisms may have in marine ecosystems. This is especially important in the Mediterranean Sea, which is a globally unique biodiversity hotspot with high diversity and endemism of flora and fauna [21].

Many studies investigating the plastisphere of floating plastics in marine environments, particularly in the Mediterranean Sea, have predominantly used amplicon-based methodologies, such as DNA metabarcoding. These studies have focused on the analysis of rRNA genes, specifically 16S for prokaryotes, 18S for eukaryotes, and ITS for fungi [12,22,23,24,25,26,27,28]. However, this methodology presents a limited capability to identify the complete spectrum of co-occurring organisms within a single sample, as each gene reflects the diversity of specific taxonomic groups. Furthermore, amplicon analysis hinders the classification of organisms at the species level. In contrast, the shotgun approach used in our study provides a distinct advantage by enabling classification at the species level, while comprehensively describing all taxa simultaneously. The exploration of eukaryotic, fungal, archaeal, and viral species within each plastisphere sample can provide valuable insights into their abundances and potential ecological roles. Moreover, investigating plastic samples collected directly from the sea can better represent natural environments compared to studies involving virgin plastics incubated in situ or in laboratory assays, as reported in many previous studies.

Here, we provide a comprehensive view of the plastisphere community composition at two sites in the northwestern Mediterranean Sea: the bays of Villefranche-sur-Mer and Toulon, in southern France. Using a shotgun metagenomic approach, we aimed to describe the full diversity of taxa that can inhabit different plastic items, and our focus extends to the often-overlooked group: viruses. They are the most abundant “lifeforms” in the oceans, influencing the composition of marine communities and playing a major role in geochemical cycles [29]. Information regarding viruses’ presence and concentration in the plastisphere could be used to inform Quantitative Microbiological Risk Assessments (QMRAs) to estimate the risks of viral infection associated with plastics [19]. In this context, it is imperative to evaluate how plastisphere organisms can interact with each other, especially viruses, and how they influence the general functioning of marine systems.

## 2. Materials and Methods

### 2.1. Sampling Sites 

**Figure 1:**
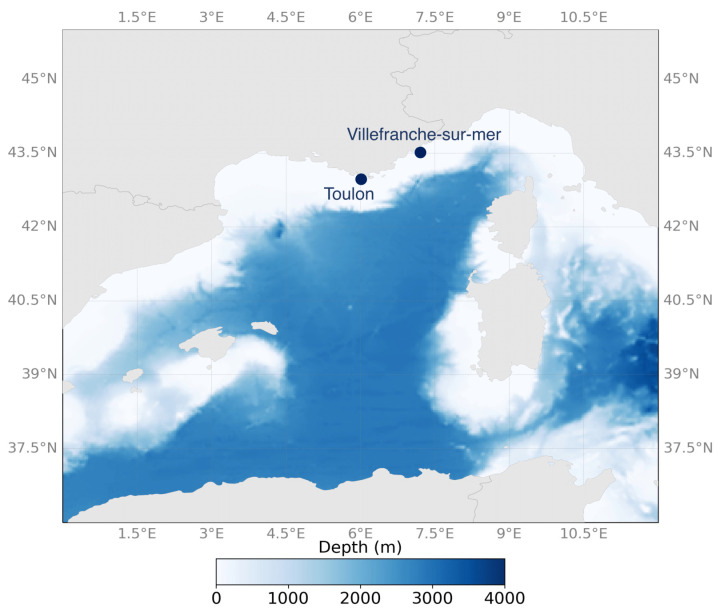
Sampling area of plastics and their associated plastisphere in the northwestern Mediterranean Sea, in the Toulon and Villefranche Bays, France. This map was created with MATLAB software version R2021b by using the *M_Map* package. Credits: Lucas Almeida.

We chose two sites in the Mediterranean Sea, the bays of Villefranche-sur-Mer (Villefranche Bay) and Toulon (Toulon Bay), in southern France (Figure 1), which are characterized by different pollution levels. Such variations depend on various factors like industrial activities, maritime traffic, urban discharges, and other environmental parameters. Although Villefranche Bay and Toulon Bay share similar climatic conditions, they experience different anthropogenic impacts. Villefranche Bay is exposed to moderate levels of pollution from the urban area of Nice [30]. At this site, the water in the bay is generally clear and salty due to the limited flow of the nearby small rivers (Roya, Var, and Paillon rivers). Fishing activity is relatively low, although the bay is a popular anchoring spot during summer for yachts and cruise vessels [31]. Conversely, Toulon Bay is subject to disturbance from direct and indirect human activities. The navy, marinas for yachts and cruise ships, shipyards, and urbanized areas around the cities of Toulon, La Seyne, and St. Mandrier contribute to these anthropogenic influences [31,32]. In addition, the historical presence of military, industrial, and port activities in Toulon has led to contamination by metals and organic compounds in the area [33,34].

### 2.2. Sampling of Plastics at Sea

Plastics of different types (fragment, foam, and film), mostly mesoplastics (5–20 mm), were collected from the sea surface either with a Manta net (330 μm, during 20 min) or directly from the seawater with sterile tweezers in the aforementioned locations. In the field, plastics were immediately placed in a sterile tube on ice, and once samples arrived in the laboratory, plastics were rinsed in sterile 0.2 μm-filtered seawater to remove weakly associated organisms (organisms co-occurring with plastics during sampling), with further storage in −80 °C until DNA extraction of the biofilm present on plastics.

### 2.3. DNA Extraction

The total DNA of the biofilm formed in eleven plastic items (nine from Villefranche Bay and two from Toulon Bay) was extracted using a Quick-DNA Fecal/Soil Microbe kit (Zymo Research) following the manufacturer’s instructions, except for the elution step, where the DNA was eluted in a lower buffer volume (30 μL) to increase DNA yield and concentration. The quality and concentration of the extracted DNA were checked with spectrophotometry using a Nanodrop (Thermo Fisher Scientific, Waltham, MA, USA).

### 2.4. Identification of Polymer Composition

After DNA extraction, plastic pieces were removed from the extraction tubes, cleaned with sterilized water, and left in the oven to dry. Fourier transform infrared (FTIR) spectroscopy analyses were carried out to identify the polymeric composition of plastics. Before analysis, all samples were dried at least 48 h at 60 °C until the absence of moisture signal in FTIR spectra. FTIR spectra were acquired using a Fourier transform infrared spectrometer (Shimadzu Corporation, model Prestige 21, Kyoto, Japan) equipped with a diffuse reflectance module. All spectra were recorded in absorbance mode using 24 scans at a spectral resolution of 4 cm^−1^ ranging from 4000 to 500 cm^−1^. Data were corrected and normalized to obtain absorbance spectra using OriginLab 8.0 software. Each spectrum was interpreted concerning the comparison against a spectral library of plastic polymers (Shimadzu IRsolution 1.5 software) and its natural weathering effect [35].

### 2.5. Assessment of the Plastisphere Composition

The extracted DNA of plastisphere samples was sent to the Eurofins Genomics Center in Germany for Next Generation Sequencing of metagenomes and taxonomic profiling. After sequencing, raw data were processed using the fastp software version 0.20.1 [36] to remove poor-quality bases from the reads (below Phred Quality 20). After quality trimming, any adapters detected in the reads were removed. Further, shorter read lengths (<30 bp) were also removed to retain only high-quality sequencing reads for each sample. In the case of paired-end reads, both sequencing reads that passed the quality control (QC) criteria were considered for downstream analysis. Read statistics are provided in Supplementary Material (Supplementary Material, Table S1).

Taxonomic profiling was performed using MetaPhlAn3 (Metagenomic Phylogenetic Analysis) [37], a computational tool for profiling the composition of microbial communities from metagenomic shotgun sequencing data, with species-level resolution. Unclassified reads were then subjected to KrakenUniq [38]. Kraken is an ultrafast metagenomic sequence classification that classifies reads by breaking each read into overlapping k-mers [39]. Each k-mer is mapped to the lowest common ancestor (LCA) of the genomes containing that k-mer in a pre-computed reference database.

To identify taxa relative abundance, read counts at various taxonomic levels (phylum, genus, and species) were considered and normalized by using the rarefy function implemented in the “vegan” bioconductor package [40]. This was performed to compare species richness from all samples in the analysis run. Rarefied read counts enabled better comparisons of Operational Taxonomic Unit (OTU) profiles between samples with different sample sizes. Abundance measured by the percentage of OTU-assigned reads from various taxonomic levels was determined and then used to generate heatmaps and bar plots at phylum, genus, and species levels. Species diversity indexes (Shannon, Simpson, Alpha diversity, and Evenness) were computed using the “vegan” package [40] in the R environment, R studio 2022.07.2_576 (R Core Team). Relative abundances of all taxa per sample could be accessed at FigShare (10.6084/m9.figshare.25233472). Principal Coordinate Analysis (PCoA) was conducted to evaluate the community composition according to polymer type considering “polyethylene” (PE) and “polypropylene” (PP), as the other polymers did not have replicates; for the plastic type (shape), we considered the categories “film” and “fragment”. The acronym VLRF in samples’ names refers to “Villefranche”, and TLN refers to “Toulon”, indicating the site where they were collected.

## 3. Results 

### 3.1. Sequencing Metrics per Plastic Sample 

We found 708,929,542 reads in the whole dataset, where 28,254,522 reads were taxonomically classified as bacteria, archaea, eukaryotes, fungi, or viruses. The remaining reads were “unclassified reads” (Supplementary Material, Table S2).

### 3.2. Taxonomic Abundance and Diversity of the Plastisphere in the Northwestern Mediterranean Sea 

**Figure 2:**
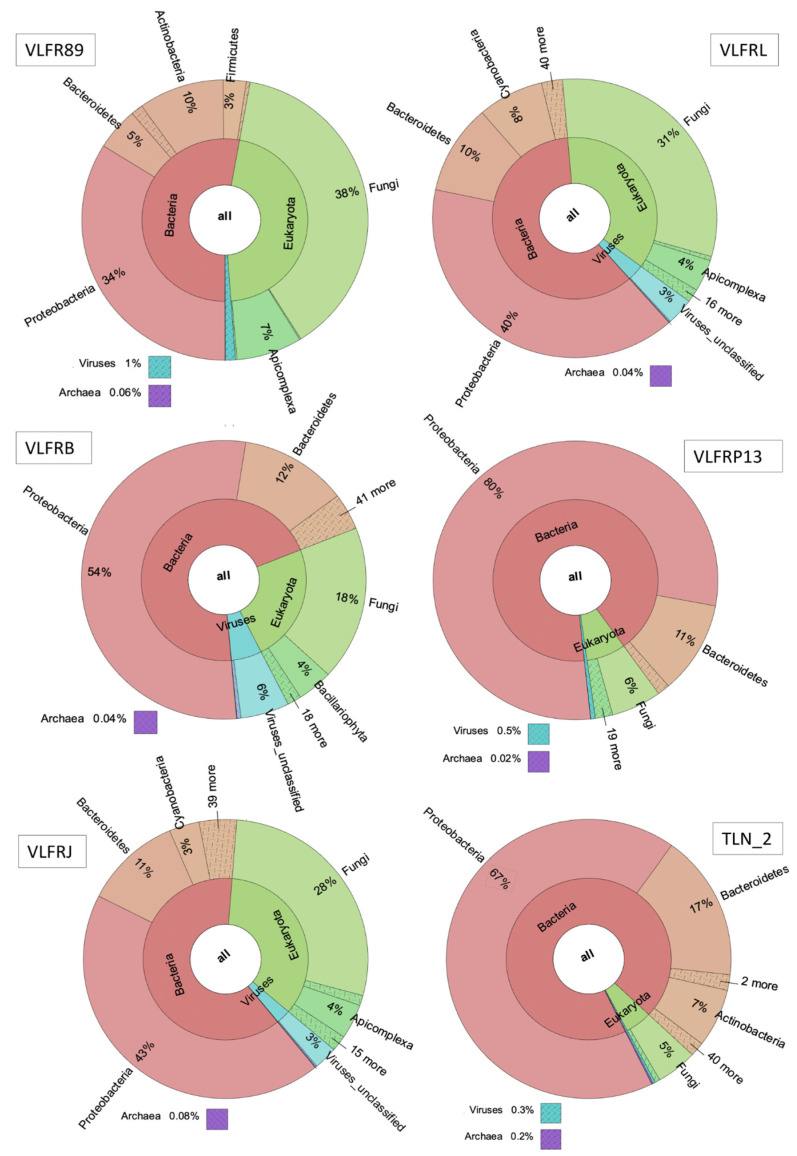
Relative abundance of taxa (bacteria, eukaryotes, viruses, and archaea) in the plastisphere of six floating plastics sampled in the northwestern Mediterranean Sea (Villefranche—VLFR, and Toulon—TLN) identified through a shotgun metagenome sequencing.

Bacteria was the most abundant kingdom observed (53 to 98% of total reads per sample), followed by eukaryotes (1% to 46% of reads per sample). Viruses’ abundance was lower compared to other kingdoms, ranging from 0.1% to 6% (Figure 2; Supplementary Material, Figure S1). Even though archaea was also present, it showed lower abundances than viruses, with a maximum of 0.2% in one sample, and ranging from 0.04% to 0.1% in the others.

**Figure 3:**
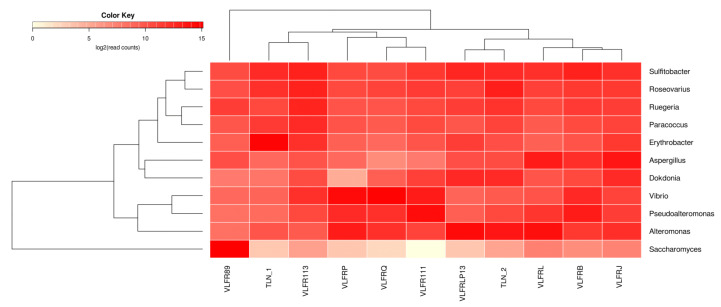
Heat map illustrating the taxonomic abundance and relationships across samples. Dendrograms, created through hierarchical clustering of abundance levels, reveal the connection between the most abundant genera (on the left) and the polymer samples (at the top). The abundance levels (number of reads associated with each taxon) are logarithmically transformed to base 2 for clarity. VLFR = Villefranche samples; TLN = Toulon samples.

**Figure 4:**
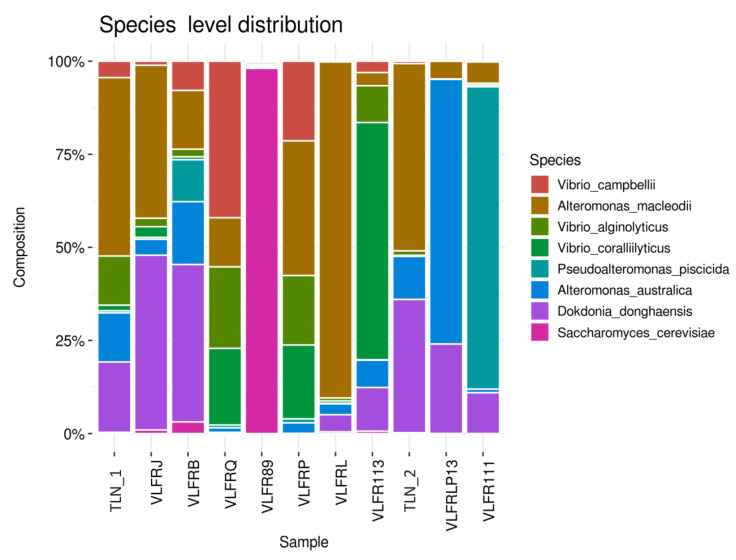
Bar plot showing the taxonomic abundance across samples. The abundance levels (number of reads associated with each taxon) are logarithmically transformed to base 2 for clarity. Taxa-level: species. VLFR = Villefranche samples; TLN = Toulon samples.

At the phylum level, Proteobacteria was the most abundant group, accounting for over 30% of reads in all samples but three, and 89% in one sample (Supplementary Material, Figure S2). Other bacterial phyla, such as Bacteroidetes, Cyanobacteria, Actinobacteria, and Planctomycetes, were among the most abundant phyla in all samples, along with Ascomytoca (fungi) and Apicomplexa (protozoan) (Figure 2; Supplementary Material, Figure S2). At the genus level, we observed a dominance of bacteria *Sulfitobacter*, *Roseovarius*, *Vibrio*, and *Ruegeria*, with variable proportions among samples (Figure 3). Other bacterial genera, such as *Erythrobacter*, *Dokdonia*, *Pseudoalteromonas*, and *Paracoccus*, as well as the fungi *Aspergillus*, were among the ten most abundant ones. The hierarchical clustering of abundance levels based on genus counts showed that one sample from Villefranche (VLFR89) was the most distant from the others (Figure 4). An explanation for this discrepancy is given hereafter, in the discussion section. It is important to highlight that the fungi *Saccharomyces* was highly abundant in this specific sample, and it was virtually absent or present in low abundances in the other samples. *Vibrio* species were particularly present in three Villefranche samples (VLFRP, VLFRQ, VLFR111), which were hierarchically clustered (Figure 3).

Notably, *Alteromonas macleodii* was the most abundant species in the entire dataset, though its prevalence varied among samples. In addition, three out of the eight most abundant species were classified as *Vibrio* species (Figure 4). Due to the high ecological importance of *Vibrio* and other potential pathogens within the marine plastisphere, we have a separate section in a subheading hereafter.

The various diversity indices based on species counts showed variances among plastisphere samples (Supplementary Material, Figure S3). Alpha diversity showed high species richness for most samples, with a lower richness in one sample from Villefranche (VLFRP13). The species evenness, however, was highly variable, with dominance of a few species in some samples, as for example sample VLFR89, which was vastly dominated by fungi *Saccharomyces cerevisiae*. Likewise, evenness was low in sample VLFRL due to the high abundance of *Alteromonas macleodii*, and also in sample VLFRP13, which presented a high abundance of the single species *Alteromonas australica*. The same occurred in sample VLFR111, where *Pseudoalteromonas piscicida* accounted for over 70% of reads (Figure 4). Although present in all samples, eukaryotes apart from fungi did not appear among the most abundant species. However, in sample VLFR89, Apicomplexa species represented 7% of reads, while in sample VLFRL, this taxon represented 4% of reads. In addition, Bacillariophyta species represented 4% of reads in sample VLFRB and 1% in sample VLFRJ.

### 3.3. Plastisphere Composition According to Polymer Type and Shape 

Our investigation into the influence of polymer type (PE vs. PP) and plastic shape (fragment vs. film) on the plastisphere community structure showed no significant impact of these factors. Principal Coordinate Analysis (PCoA) shows that neither the polymer type (PERMANOVA, F = 1.5864, *p* = 0.19) nor the plastic shape (PERMANOVA, F = 2.8392, *p* = 0.09) shaped the structure of the plastisphere (Supplementary Material, Figure S4), emphasizing the complex and dynamic nature of these microbial communities in response to environmental factors.

### 3.4. Viruses in the Mediterranean Plastisphere 

Although viruses were present in all samples, their relative abundances were low compared to bacteria and eukaryotes. The number of viral reads per sample ranged from 651 to 13,611, and the majority of viruses were “unclassified_viruses”. From the classified viruses, Herpesvirales exhibited significant abundances: 22% in sample VLFRL, 18% in sample VLFRJ, and 14% in sample VLFRB, with lower abundances in the remaining ones. In addition, the Caudovirales order showed abundances of 6% in sample VLRFB, and 1% in samples VLFRJ and VLFRL, being less representative in the others. Furthermore, the Poxviridae family (which includes *Cowpox* and *Taterapox* viruses) represented 43% of viruses in one sample from Villefranche (VLFR89), but its abundance was lower in the remaining samples. 

**Figure 5:**
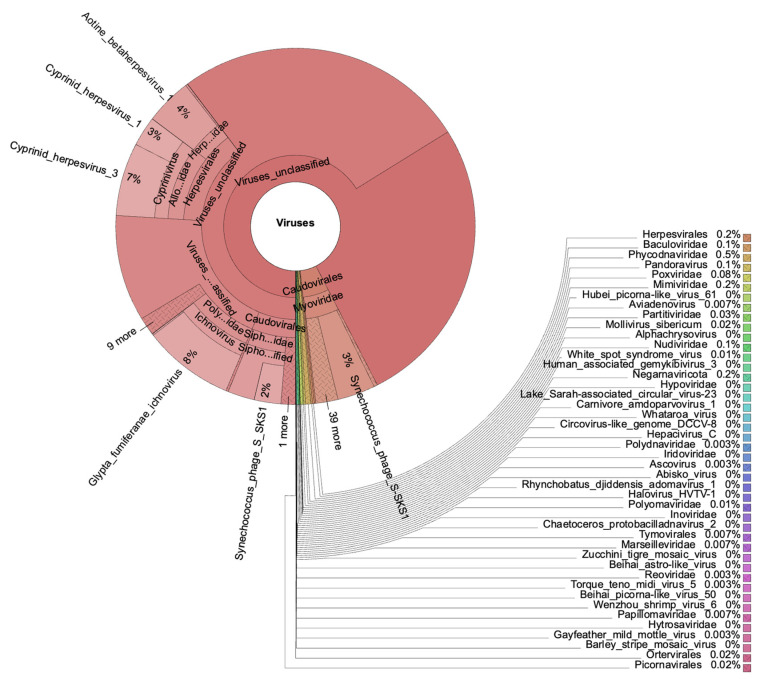
Relative abundance of viruses detected in one plastisphere sample (VLFRB) collected in the coastal area of Villefranche-sur-Mer, southern France, to illustrate the diversity of viral groups inhabiting one plastic item drifting in the northwestern Mediterranean Sea.

We detected the White Spot Syndrome virus (WSSV), a shrimp penaeid pathogen, in samples from both sites. WSSV abundances were low in all samples, representing a maximum of 0.2% of viral reads. We also identified the *Wenzhou Shrimp* virus in one sample from Villefranche (VLFR113), with 0.08% abundance compared to other viruses. *Cowpox* species were present in all samples but one, representing 3% of viruses in sample VLFRL. Moreover, *Taterapox* sp. was present in eight out of eleven samples, accounting for 1% of viruses in sample TLN_2 and ranging from 0.04 to 0.6% in the remaining ones. Like *Taterapox*, the *Zika* virus was also present in eight samples, with a higher relative abundance of 2% in sample VLFR113. Baculoviridae species were observed with abundances up to 0.8%. The Phycodnaviridae family represented up to 5% of all viruses in one sample. The remaining viruses presented lower abundances within the whole dataset (Figure 5).

### 3.5. Other Plastisphere Organisms with Important Ecological Functions 

Three *Vibrio* species (*V*. *campbelli*, *V*. *alginolyticus*, and *V*. *coralliilyticus*), which are emerging pathogens for aquatic organisms [41,42], were among the eight most abundant species in the whole dataset. In addition, *Staphylococcus* species, such as *S*. *aureus*, which is one of the most important pathogens found in seafood [43], were also present, with *S. aureus* representing 3% of Bacilli bacteria in sample VLFR89.

The fungi *Aspergillus lentulus* was observed in all samples, representing up to 16% of fungal reads in one sample (VLFRLP13) and 3% of reads in samples VLFRL and VLFRJ. The relative abundance of *A. lentulus* was lower in samples from Toulon (≤0.3%). In addition to fungi, Bacillariophyta (diatoms) was another frequent and abundant eukaryotic group, mainly the species *Thalassiosira pseudonana* and *Phaeodactylum tricornutum*. Moreover, eukaryotes belonging to the apicomplexa taxon, which are parasites of animals and, in some cases, of humans, were also observed. One highly abundant apicomplexa species, *Toxoplasma gondii*, represented 6% of total reads in sample VLFR89 and over 20% of the apicomplexa reads in samples VLFR111, VLFR113, and VLFRQ. In addition, cyanobacteria, including benthic species, accounted for 19% of reads in one sample (VLFRP), and ranged from 0.2 to 8% of total reads in the other samples. Nostocales, Oscillatoriales, and Synechococcales were the most abundant orders of cyanobacteria in all samples, alongside “unclassified cyanobacteria”. From the Nostocales order, *Calothrix* species stood out as the most abundant group.

Numerous microorganisms with the potential to biodegrade plastics were also found in our samples, some of them among the ten most abundant species, such as those bacteria identified as belonging to *Alteromonas* and *Pseudoalteromonas* genera. We also observed *Alcanivorax borkumensis* and *Alcanivorax jadensis* as highly frequent, the latest representing 2% of reads in one sample from Toulon. In addition, bacteria *Pseudomonas stutzeri* and *Oleibacter marinus*, as well as fungi from *Aspergillus* genus and bacteria from *Erythrobacter* genus, were also present in all samples.

## 4. Discussion

Our analyses provide original information on the plastisphere of the northwestern Mediterranean Sea. We have gone a step further compared to amplicon studies (rRNA 16S and 18S, or ITS2 region) and described the full diversity of micro-life present in different plastic samples collected from the sea, showing their relative abundances as a function of polymer type, plastic shape, and sampling sites. We also described an undervalued and ecologically important taxon: viruses. It is pivotal to have a panorama of all species living together in a single plastic item to understand how they interact with each other, as well as with the surrounding environment. The shotgun approach offers a distinctive advantage by allowing affiliation down to the species level. This resolution facilitates a more precise identification of the ecological role of plastisphere members.

### 4.1. Full Diversity and Potential Ecological Role of the Mediterranean Plastisphere

The dominance of bacteria over other kingdoms, which is not possible to determine with amplicon-based approaches, is in accordance with the few previous studies worldwide [6]. Bacteria’s success in marine environments allows them to rapidly colonize artificial surfaces in contact with seawater [44]. In addition, their association with eukaryotic organisms, such as (micro)algae and invertebrates, which are indeed common plastisphere inhabitants [17,18], would explain their dominance. Fungi are also common members of the marine plastisphere, even though they have not been well-evaluated, despite their non-trivial ecological roles in marine environments [45,46,47].

Like in other environments, many ecological interactions can take place in the plastisphere, such as commensalism, parasitism, mutualism, predation, symbiosis, competition, and nutrient cycling [17]. The huge diversity of taxa we found harbored in individual plastic items highlights the potential for diverse ecological interactions within epiplastic communities. For example, we can expect symbiotic relationships between bacteria and microalgae. Various studies have identified Proteobacteria and Bacteroidetes as the predominant bacterial phyla associated with diatoms, and high frequencies of the genera *Sulfitobacter*, *Alteromonas*, and *Flavobacterium* have been consistently observed in these associations [48,49,50]. Indeed, diatoms were present in all samples, mostly species from the genuses *Thalassiosira* and *Phaeodactylum*, alongside the three aforementioned bacteria genera. The co-occurrence of these groups in the same samples opens the floor for a more in-depth investigation of how plastisphere organisms interact with each other. 

The most abundant species in our dataset, *Alteromonas macleodii*, is a heterotrophic bacterium cosmopolitan in the oceans that utilizes various substrates for growth [51]. *A*. *macleodii* is able to bloom under high nutrient concentrations and plays a particularly relevant role in ecological carbon cycling in the ocean [51]. This bacterium exhibits a high tolerance to heavy metals and is among the early colonizers of copper-based substrates, such as ship paints [52,53]. Considering plastics’ additives, as well as their ability to adsorb metals from the seawater [54], this could explain the high amount of *A. macleodii* in our plastisphere samples, especially in one sample from Toulon, which is a highly polluted area [34].

Biofilm formation on microplastics can have an impact on the cycling of carbon and nitrogen in the ocean [55,56]. Species from *Thioclava* genus, a Rhodobacteraceae (Proteobacteria) group highly abundant in our samples, play an important role in the material cycle and energy flow, particularly in carbon dioxide fixation [57] and nitrate reduction [58]. In addition, we found numerous species of Planctomycetes, a bacterial phylum widely distributed in aquatic and terrestrial habitats, which play a considerable role in the global cycle of metals and nitrogen [59]. This group was also detected in the marine plastisphere of the South Atlantic [18] and South Pacific [60] Oceans, thus reinforcing the need to understand its role in biogeochemical cycles as a plastisphere member. Moreover, *Roseovarius*, another highly abundant and ubiquitous group we observed, comprises bacteria species known for their involvement in the sulfur cycle [61], as well as for its ability to degrade complex organic compounds [62]. 

It has recently been claimed that cyanobacteria-colonizing plastics use a completely different light-harvesting mechanism compared to cyanobacteria from seawater. They perform photosynthesis through phycobilisome complexes, whereas in the surrounding seawater, photosynthesis takes place mainly in the chlorophyll-binding complexes [5]. Phycobilisome proteins act as nitrogen reservoirs, increasing cyanobacteria’s viability in nitrogen-limited environments on plastic surfaces [5]. This phenomenon could be more important in oligotrophic areas such as the Mediterranean Sea [22], and also in the middle of the ocean basins, where we find gyres that accumulate plastic waste [63].

Another bacterium found among the eight most abundant species in our samples, *Dokdonia donghaensis*, belongs to a genus that comprises strictly biofilm-forming marine Flavobacteriaceae. This bacterium has been found in higher abundances on plastics compared to seawater and marine sediments from Naples, Italy [25], and was correlated with one metal resistance gene (czcA) on biocidal-based antifouling coatings [64]. Basili et al. [25] suggested that the plastic surface offers more advantageous conditions for the survival and growth of these microbes compared to the aquatic environment. The dominance of *Saccharomyces cerevisiae* in one sample (VLFR89) raises the question of that piece of plastic being recently egested by a living organism, as *S. cerevisiae* is not commonly found in the seawater or marine sediments, but is common in gut and stomach contents of marine fish and invertebrates [65]. 

The species identified as having the capacity for plastic degradation, notably *Alcanivorax* and *Pseudomonas* bacteria, alongside *Aspergillus* fungi, are consistent with reports of their presence in the global marine plastisphere. They have been documented as possessing the potential to degrade different plastics, including low-density polyethylene—LDPE [66,67], the dominant polymer in our samples. Additionally, *Oleibacter marinus* and *Erythrobacter* sp. have been recognized for their capability to degrade hydrocarbons [68,69]. Despite the identified organisms exhibiting the potential to utilize plastics as a carbon source, it is important to consider that the marine environment offers alternative energy sources that are more readily assimilated than synthetic polymeric chains. Consequently, the biodegradation of plastics in natural conditions could be a slow process [69], which explains their persistence in the ocean. 

It is uncertain whether plastics coming from areas more exposed to contamination have more pathogens. If true, it raises concerns about numerous Mediterranean coastal zones affected by human activities, prompting inquiries into associated health risks. We did not conduct location-specific statistical analyses due to the limited sample size from Toulon. Despite the higher pollution levels in Toulon, the viral presence in Toulon samples did not exceed the abundance of viruses in Villefranche samples. Notably, samples from Villefranche Bay showed a higher relative abundance of viruses and pathogenic bacteria, especially *Vibrio* species. 

### 4.2. Viruses in the Marine Plastisphere

Viruses were discovered in the nineteenth century and they used to be seen as agents causing infectious diseases [70]. However, in the last decade, this domain has had its role recognized in host regulation and the maintenance of natural ecosystems [71]. Therefore, in addition to the pathogenic functions of certain species, a vast variety of viruses have been found in diverse environmental samples, where they represent a large reservoir of unexplored genetic diversity [29]. Viruses control the abundance of bacteria and other microorganisms, as well as the transfer of genes between different species, thus playing a role in the natural balance of microbial communities [72]. 

To date, studies on viruses in the plastisphere remain limited, with their ecological roles being largely unexplored [20,73,74]. Here, we report for the first time the White Spot Syndrome virus (WSSV) as a member of the marine plastisphere. WSSV can cause a viral infection that affects penaeid shrimps. This highly lethal and contagious disease has caused devastating outbreaks in shrimp farm populations in the Mediterranean coastline of Europe–Greece, Italy, Spain, and Turkey [75], as well as in Brazil [76], Thailand [77], and India [78]. Lightner et al. [79] reported a cumulative economic loss of USD 15 billion worldwide over two decades due to WSSV outbreaks since its first report in China in 1991. Another shrimp pathogen, the *Wenzhou shrimp* virus [80], was also present in one sample from Villefranche Bay.

In addition, we observed *Baculovirus* spp. in our samples, which can be pathogens for arthropods and infect crabs and shrimps by attacking their hepatopancreas [81]. The adhesion of viruses to their hosts, which include many plastisphere organisms, is of concern. Some plastics float and are dispersed in the oceans; thus, they could spread their associated organisms across regions. This could lead to the introduction or spread of infections from coastal aquaculture systems, potentially causing ecological and economic damage.

Other groups of marine organisms, such as mammals, can be affected by viruses. *Poxvirus* has been associated with skin lesions in bottlenose dolphins (*Tursiops truncatus*), killer whales, long-beaked common dolphins (*Delphinus capensis*), and Burmeister’s porpoises (*Phocoena spinipinnis*) [82]. Cutaneous *Poxvirus* infections in cetaceans can develop on any part of the body and cause the so-called “tattoo skin disease–(TSD)” [83]. In addition, the Phycodnaviridae family (which represented 5% of all viruses in one sample), comprises members known to infect marine and freshwater algae [84]. They can have various pathological effects depending on the viral species and the host, and play important roles in the algal community structure and dynamics of algal blooms [85].

Moresco et al. [20] recently found an infectious virus associated with biofilm-colonizing microplastics in a eutrophic lake. They suggested that the interaction between the virus and the biofilm enhances virus survival compared to when it is in the water phase, as the presence of a protective biofilm on microplastic surfaces can potentially increase virus stability and dissemination. Lu et al. [73], investigating the adsorption capacity of viruses on microplastics (in lab assay), confirmed this assumption. They showed that a maximum of 65.9 ± 1.9% of viruses retained their infectivity after adsorption on pristine or UV-aged microplastics, which prolonged their survival. Although freshwater and seawater environments are different, it is important to consider the impacts of plastisphere viruses in the ocean. Indeed, we have shown here that viruses are frequent in, at least, all our plastisphere samples, highlighting their occurrence in this new ecosystem. 

In a recent review, Moresco et al. [19] suggested that the ability of viruses to bind to bacterial biofilm may be influenced by specific microbial community characteristics, such as the succession stage and bacterial diversity. Our analysis focused on plastics collected from the ocean, revealing various species in the Mediterranean plastisphere, including the WSSV as a novel member of the marine plastisphere. This knowledge about viral species associated with plastics in the ocean could guide future research to assess their activity under different biofilm conditions. The co-occurrence of various species in each of our plastisphere samples opens the floor for detailed investigations into potential interactions among viruses and other plastisphere members. We also found the *Synechococcus phage* virus in all samples. Cyanophages play an important role in mediating cyanobacterial diversity, distribution, and nutrient cycling, besides their influence on cyanobacterial evolution [86]. Synechococcales was one of the most abundant orders of cyanobacteria in our dataset, so it is not surprising to also find this virus. Therefore, the presence of viruses in our plastisphere samples may be related to their interaction with other plastisphere organisms. 

The *Human herpesvirus 3* has been identified in microplastics from the Bei-Lun River, located on the Sino–Vietnamese border, and Caudovirales and Phycodnaviridae were also common viral groups within the plastisphere at this site [74]. Polypropylene and polyethylene, which were also the most prevalent polymer types we evaluated here, exhibited a higher environmental risk associated with viruses compared to other polymers, including the transport of antibiotic-resistant genes (ARGs) and virulence factors (VFs) [74]. These authors evaluated the up and downstream parts of the river over 109 km and found that the diversity of viral species detected in polypropylene samples surpasses that in surface waters and sediments, suggesting that microplastics serve as long-distance carriers for viruses in rivers.

In natural environments, both humans and animals inevitably encounter plastics with their associated plastisphere. This proximity creates opportunities for genetic exchange and elevates the risk of host-specific gene transfer between unrelated pathogens, potentially leading to the emergence of novel pathogen strains capable of colonizing new hosts [87]. The plastisphere offers protection against environmental factors, for example, UV radiation, which may influence the development of resistance mechanisms in some viruses [87].

Although little is known about where pathogenic viruses occur outside of their host or their modes of transmission, there is a great understanding that some viruses can have a wide host range and circulate between marine and freshwater areas [29]. In this sense, the transport of plastics and the viruses they might carry could increase viruses’ dispersion between different oceanic zones, as well as between oceans and continents. Despite the fact that we found viruses in lower abundances (40.504 reads in total) compared to bacteria and eukaryotes, it might raise attention. Even a low infectious dose of some pathogens can successfully cause dangerous infection, and biological substances adsorbed on plastics might be of large concern for human and animal health [73].

### 4.3. Other Potential Pathogens within the Mediterranean Plastisphere

The presence of potentially human-pathogenic organisms in the plastisphere of aquatic environments is a subject of much debate [88]. Despite the current lack of consensus, the potential impacts of bacteria and viruses within the marine plastisphere deserve to be taken into account, especially considering its role in ecosystem dynamics and aquatic health.

Keswani et al. [89] highlighted the risks of human exposure when swimming in plastic-contaminated waters and its associated pathogens. More recently, Mincer et al. [90] identified pathogenic genes in the plastisphere by evaluating Metagenome-Assembled Genomes and cultures derived from plastisphere *Vibrio* spp., reinforcing the role of marine plastic debris in harboring and promoting favorable conditions for pathogens to evolve. In addition, when evaluating metagenomes and metaproteomes from the marine plastisphere, Messer et al. [7] also found several expressed proteins associated with virulence factors, such as *Pseudoalteromonas* lipase, *Lysobacter* mycobactin siderophore (Phenyloxazoline synthase MbtB), and *Streptomyces* toxins (papain fold toxin domain, Ntox27 domain-containing protein).

Among *Vibrio* species, only some are indeed pathogenic. With the advantage of having accessed the plastisphere through shotgun sequencing, we observed potential animal and human pathogens as the most abundant taxa, for example, *V*. *alginolyticus*, commonly found in marine environments and medically important for causing ear, eye, and wound infections in humans [91,92,93]. This species is also present in the bodies of aquatic animals such as pufferfish, where it is responsible for producing toxins like tetrodotoxin (TTX) [94]. This endosymbiotic bacterium in pufferfish is often passed down the food chain and the ingestion of contaminated pufferfish is a common route of toxicity responsible for human intoxications [94]. 

*Vibrio coralliilyticus*, another highly abundant species in our dataset, has been shown to be virulent to corals [95], hence its name. It also poses a threat to both the Pacific and Eastern Oysters (*Cassostrea gigas* and *C. virginica*) [96], as well as to the rainbow trout (*Oncorhynchus mykiss*) [97]. This bacterium is of great interest due to its direct contribution to coral disease outbreaks, a concern for the already vulnerable group facing bleaching caused by rising sea temperatures due to climate change, pollution, and other causes. *V. coralliilyticus* poses a risk to coral reefs and also impacts aquaculture, where it can lead to significant mortalities in larval oyster hatcheries [96]. Naudet et al. [98] recently investigated the potential role of plastics in aquaculture as a support for bacterial biofilm that can include potential human pathogenic bacteria (PHPB) and antibiotic-resistant bacteria (ARB). They found that isolates from aquaculture plastics showed higher significant multiple antibiotic resistance (MAR) compared to seawater samples, sediments, and fish guts, and suggested that plastics act as a reservoir of PHPB and ARB in aquaculture, potentially threatening the health of farmed fish and human consumers [98].

*Vibrio parahaemolyticus*, an animal/human pathogen that can live associated with plastics [99], exhibits increased virulence above 27 °C [100]. Recently, this bacterium was found attached to synthetic microfibers floating in the Mediterranean Sea [27] in a location where the sea temperature during the summer of 2022 reached around 30 °C with the European heat wave [101]. Similar to many bacteria species, the virulence of *V*. *coralliilyticus* is temperature-dependent, with the highest virulence observed above 27 °C [102]. This phenomenon, which could become recurrent, may favor the proliferation and expression of bacterial virulence, potentially amplifying the impact of pathogens in the Mediterranean Sea.

*Pseudoalteromonas piscicida*, another highly abundant bacteria found in the sampled mesoplastics from this study, was previously identified as a pathogen that causes bleaching disease in commercially farmed brown algae *Saccharina japonica* [103]. In addition, Planctomycetes, traditionally recognized as an environmental bacteria, have recently been linked to human pathology as opportunistic pathogens, arousing a great interest for clinical microbiologists [104]. Moreover, numerous apicomplexa species, such as *Toxoplasma gondii*, which was abundant in our samples, are significant pathogens for animals and humans [105]. Unlike bacteria, these parasites share metabolic pathways with their hosts, which makes drug development challenging. Targeting them without harming the host is a complex issue [105,106]. 

Research on the interaction between nanoplastics (NPs) and microorganisms is still limited. It is suggested, however, that nanoplastics cannot induce the formation of the plastisphere but instead form an eco-corona [107,108], a unique layered structure formed by the interaction of NPs and natural organic matter (e.g., proteins, carbohydrates, etc.) [109], which is beyond the scope of our study.

## 5. Conclusions

This study is a comprehensive characterization of the marine plastisphere from floating plastics sampled in the northwestern Mediterranean Sea. We found a great diversity of taxa living in different plastic types, with communities dominated by bacteria and numerous eukaryotes, especially fungi. Viruses were also frequent members of the Mediterranean plastisphere, being present in all samples, albeit at lower abundances compared to bacteria and eukaryotes. Our exploration revealed numerous species with potential implications for animal and human health, some identified for the first time in the marine plastisphere, such as the penaeidae pathogen White Spot Syndrome virus (WSSV). The simultaneous presence of symbiotic bacteria and their hosts (e.g., microalgae) in certain samples provides an opportunity to assert that these groups are in association with the plastisphere. The physico–chemical characteristics of plastics, i.e., the polymer composition and shape, did not influence the structure of the plastisphere communities, which is in line with other studies carried out worldwide. Furthermore, we observed that numerous species in the plastisphere are integral components of biogeochemical cycles. As we cannot manage what is not known, it is imperative to evaluate what inhabits plastics that are already in the sea to comprehend the interactions among these organisms, and ultimately how plastics and their associated communities influence the surrounding seawater and global oceanic processes. 

Future studies in health management must prioritize assessing viral presence across various oceanic regions to track the distribution of viral groups in the plastisphere, particularly pathogens, with global ecological implications. Additionally, detailed investigations are essential to understand the environmental conditions under which potential pathogens within the marine plastisphere become active and pose risks to marine wildlife and humans, especially in light of increasing temperatures at sea due to climate change. Furthermore, research on the role of plastisphere organisms in biogeochemical cycles, such as diazotrophic bacteria, particularly on plastics found in natural environments, is crucial to determine their impact on the functioning of the global ocean.

## Data Availability

Data are contained within the article and supplementary material.

## Supplementary Materials

The following supporting information can be downloaded at: https://www.mdpi.com/article/10.3390/microorganisms12030444/s1. Table S1: Sequence Quality Metrics overview; Table S2: Number of reads per sample according to sampling site, and plastic categories; Figure S1: Relative abundance of taxa (Bacteria, Eukaryote, Viruses and Archaea) identified through a next generation metagenome sequencing; Figure S2: Bar plot showing the taxonomic abundance across samples; Figure S3: Diversity indices (Simpson, Shannon, InvSimpson, Alpha, Species No and Evenness) based on species counts; Figure S4: Principal Coordinate Analysis (PCoA) based on the Jaccard distance matrix showing the community composition.
