# Supplementary material for: Assessing the Plastisphere from Floating Plastics in the Northwestern Mediterranean Sea, with Emphasis on Viruses
Source: Microorganisms. 2024 Feb 22;12(3):444. doi: 10.3390/microorganisms12030444 (PMC10972446; doi:10.3390/microorganisms12030444)
Supplement: Supplementary file 1 [file microorganisms-12-00444-s001.zip › microorganisms-2835491-supplementary.pdf]

microorganisms

MDPI

Supplementary Material

Assessing the Plastisphere from Floating Plastics in the Northwestern Mediterranean Sea, with Emphasis on Viruses

Ana Luzia Lacerda [1,*], Jean-François Briand [2], Véronique Lenoble [3], Eliézer Quadro Oreste [4], Felipe Kessler [4] and Maria Luiza Pedrotti [1]

* Correspondence: ana.luzia-lacerda@imev-mer.fr

Table S1. Sequence Quality Metrics overview. For each sample, the following Quality Control metrics are provided. Sample Name: name of the sample. Total Raw Reads: the total number of raw sequencing reads generated for the sample. Total HQ Reads: the total number of high quality reads after sequence cleaning and filtering. HQ Bases (Q30): Percentage of high quality bases having at least phred quality 30. GC Content: GC content in percentile of high quality sequencing reads. Mean Read Length (bp): Average read length in bp of high quality sequencing reads. HQ Reads%: High Quality Reads percentage.

Sample ID	Total raw reads	Total HQ reads	HQ Bases (Q30)	GC content	Mean read length (bp)	HQ reads%
**VLFR89**	11.14 M	10.94 M	91.1%	50.7%	147	98.2%
**VLFR111**	57.36 M	56.52 M	89.9%	52.5%	149	98.5%
**VLFRB**	82.18 M	80.84 M	93.3%	43.0%	147	98.4%
**VLFRQ**	117.35 M	115.91 M	93.6%	52.2%	148	98.8%
**TLN_1**	76.61 M	75.56 M	93.1%	56.3%	149	98.6%
**TLN_2**	68.96 M	68.08 M	93.0%	52.6%	148	98.7%
**VLFRL**	80.37 M	78.47 M	92.6%	39.8%	147	97.6%
**VLFRJ**	58.67 M	57.38 M	92.4%	42.5%	148	97.8%
**VLFR113**	50.37 M	49.78 M	92.3%	48.9%	148	98.8%
**VLFRLP13**	45.29 M	44.14 M	90.3%	44.9%	148	97.4%
**VLFRP**	72.09 M	71.32 M	93.7%	46.9%	149	98.9%

Table S2. Number of reads per sample (Sample ID), according to sampling site (VLFR = Villefranche bay; TLN = Toulon bay), polymer composition (PE = polyethylene, PP = polypropylene, PS = polystyrene, EVA = Ethylene vinyl acetate), type (fragment, film, foam), and size class (microplastics < 5 mm, and mesoplastics 5–200 mm in length).

Sample ID	Reads	Classified Reads%	Unclassified Reads%	Polymer	Type	Size class
**VLFR89**	10936806	4.74%	95.26%	PE	Film	Mesoplastic
**VLFR111**	56521770	5.65%	94.35%	PS	Foam	Microplastic
**VLFRB**	80840978	2.21%	97.79%	PP	Film	Mesoplastic
**VLFRQ**	115905456	6.25%	93.75%	EVA	Film	Mesoplastic
**TLN_1**	75562644	6.02%	93.98%	PE	Film	Mesoplastic
**TLN_2**	68075400	3.77%	96.23%	PE	Film	Mesoplastic
**VLFRL**	78469264	1.94%	98.06%	Plastic	Fragment	Mesoplastic
**VLFRJ**	57384128	1.67%	98.33%	Plastic	Fragment	Mesoplastic
**VLFR113**	49776966	2.22%	97.78%	PE	Film	Mesoplastic
**VLFRLP13**	44136156	3.73%	96.27%	PP	Film	Mesoplastic
**VLFRP**	71319974	4.44%	95.56%	PP	Film	Mesoplastic

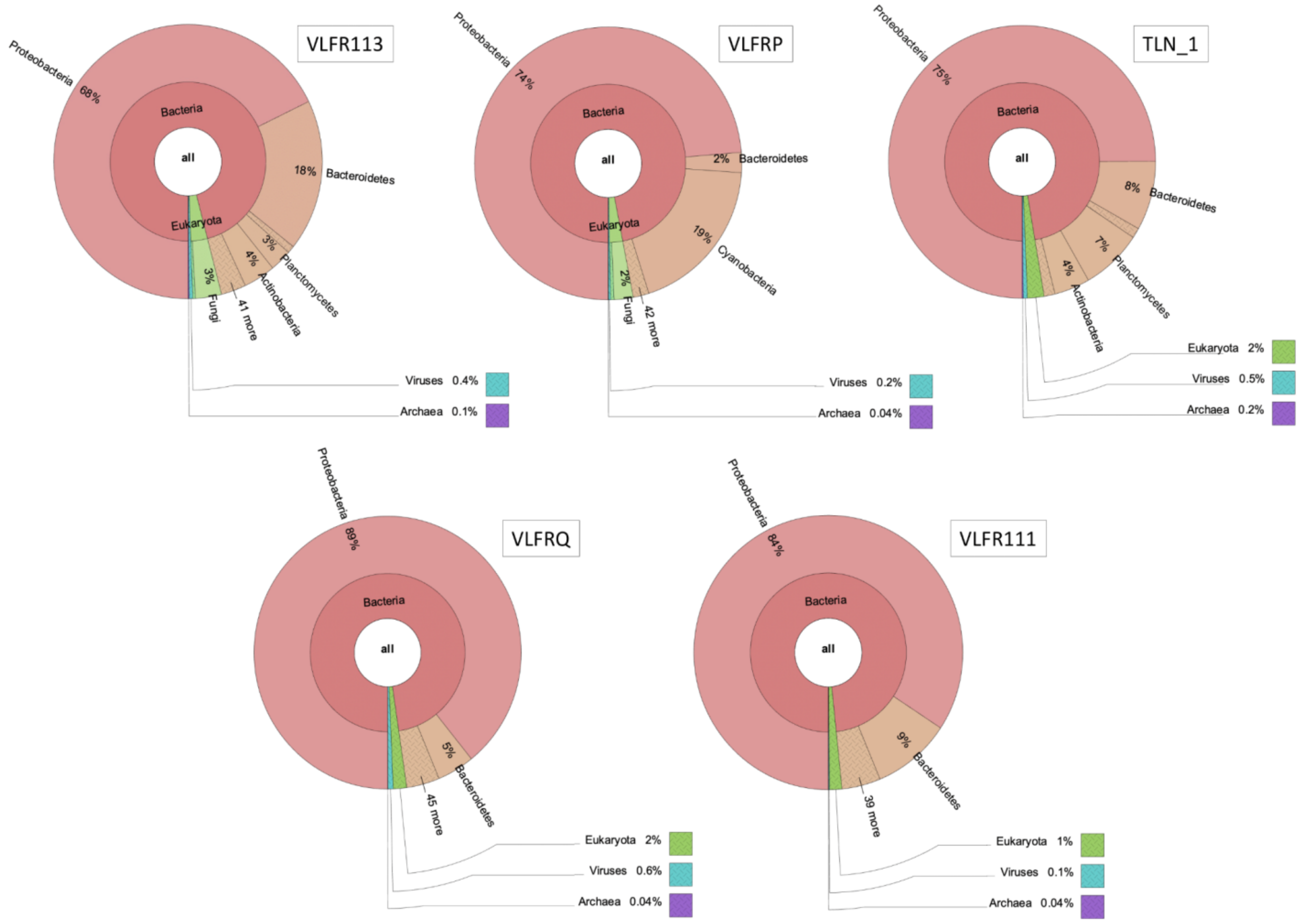

Figure S1. Relative abundance of taxa (Bacteria, Eukaryote, Viruses and Archaea) in the plastisphere of five floating plastics sampled in the northwestern Mediterranean Sea (Toulon – TLN and Villefranche – VLFR), identified through a next generation metagenome sequencing.

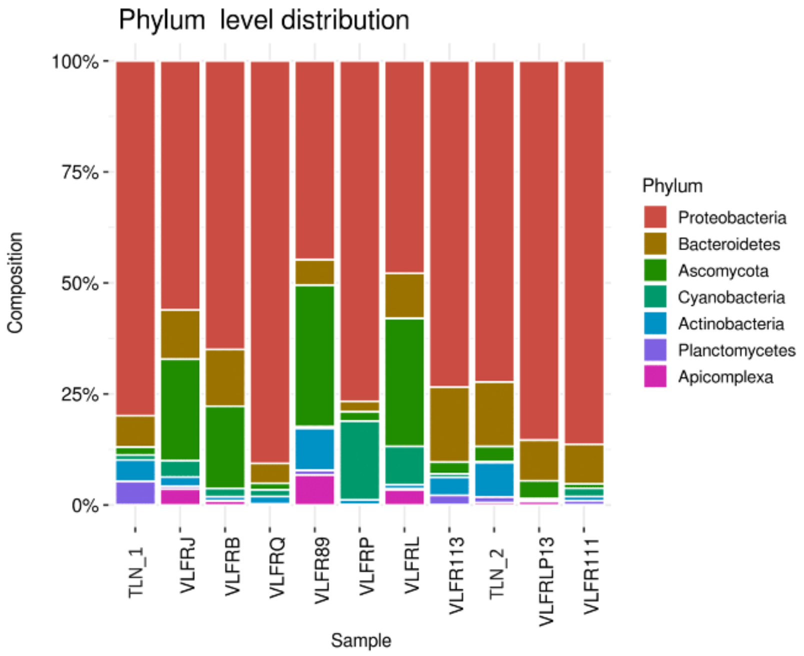

Figure S2. Bar plot showing the taxonomic abundance across samples. The abundance levels (number of reads associated with each taxa) are logarithmically transformed to base 2 for clarity. Taxa-level: Phylum.

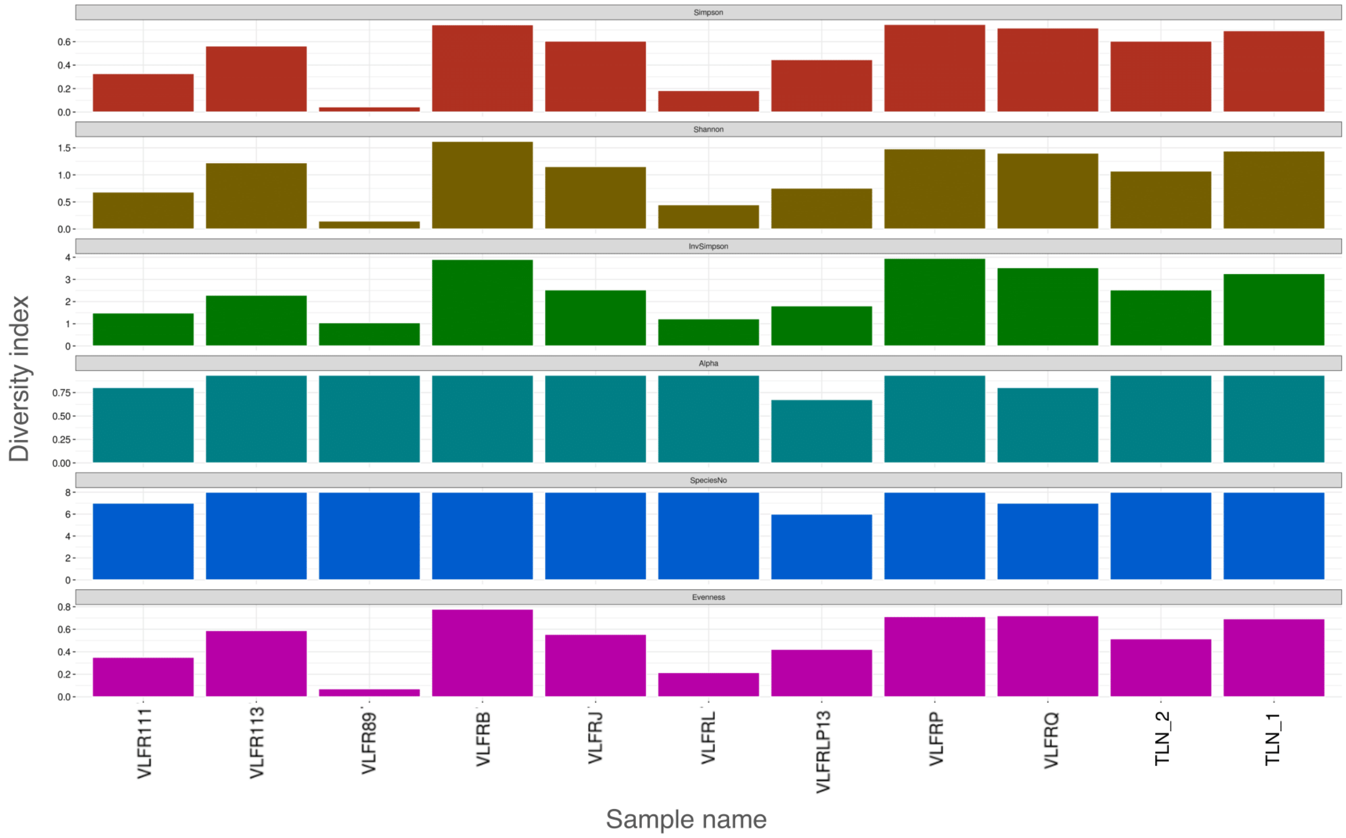

Figure S3. Diversity indices (Simpson, Shannon, InvSimpson, Alpha, SpeciesNo and Evenness) based on species counts from the coastal plastisphere in the northwestern Mediterranean Sea. VLFR = Villefranche, TLN = Toulon.

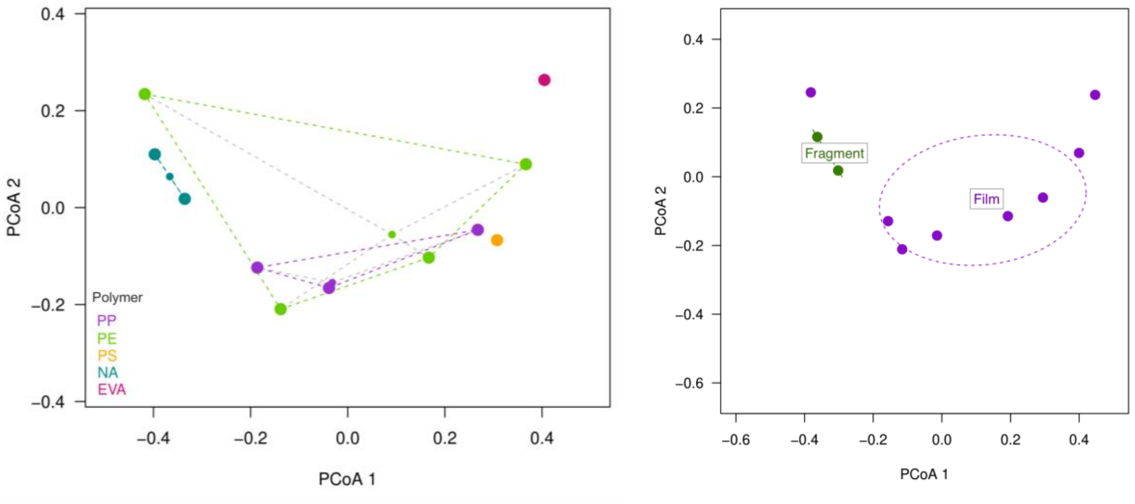

Figure S4. Principal Coordinate Analysis (PCoA) based on the Jaccard distance matrix showing the community composition of the plastisphere in the northwestern Mediterranean Sea according to polymer type (**left**) and shape (**right**).
